# Omentin: a biomarker of cardiovascular risk in individuals with axial spondyloarthritis

**DOI:** 10.1038/s41598-020-66816-x

**Published:** 2020-06-15

**Authors:** Fernanda Genre, Javier Rueda-Gotor, Sara Remuzgo-Martínez, Verónica Pulito-Cueto, Alfonso Corrales, Verónica Mijares, Leticia Lera-Gómez, Virginia Portilla, Rosa Expósito, Cristina Mata, Ricardo Blanco, Javier Llorca, Vanesa Hernández-Hernández, Esther Vicente, Cristina Fernández-Carballido, María Paz Martínez-Vidal, David Castro-Corredor, Joaquín Anino-Fernández, Carlos Rodríguez-Lozano, Oreste Gualillo, Juan Carlos Quevedo-Abeledo, Santos Castañeda, Iván Ferraz-Amaro, Raquel López-Mejías, Miguel Á. González-Gay

**Affiliations:** 1 Research group on genetic epidemiology and atherosclerosis in systemic diseases and in metabolic diseases of the musculoskeletal system, IDIVAL, Santander, Spain; 2 Rheumatology Division, Hospital Comarcal de Laredo, Laredo, Spain; 3 Department of Epidemiology and Computational Biology, School of Medicine, University of Cantabria, and CIBERESP, IDIVAL, Santander, Spain; 4 Rheumatology Division, Hospital Universitario de Canarias, Santa Cruz de Tenerife, Spain; 5 Rheumatology Division, Hospital Universitario de La Princesa, IIS-Princesa, Madrid, Spain; 6 Rheumatology Division, Hospital Universitario de San Juan, Alicante, Spain; 7 Rheumatology Division, Hospital General Universitario de Alicante, Alicante, Spain; 8 Rheumatology Division, Hospital General Universitario de Ciudad Real, Ciudad Real, Spain; 9 Rheumatology Division, Hospital Universitario de Gran Canaria Dr. Negrín, Las Palmas de Gran Canaria, Spain; 10 SERGAS and IDIS, The NEIRID Group, Santiago University Clinical Hospital, Santiago de Compostela, Spain; 11 School of Medicine, University of Cantabria, Santander, Spain

**Keywords:** Biological techniques, Molecular biology, Biological techniques, Molecular biology, Biological techniques

## Abstract

Cardiovascular (CV) disease is the main cause of mortality in axial spondyloarthritis (axSpA). CV risk is enhanced by dysregulation of adipokines. Low omentin levels were associated with metabolic dysfunction and CV disease in conditions different from axSpA. Accordingly, we evaluated the genetic and functional implication of omentin in CV risk and subclinical atherosclerosis in a cohort of 385 axSpA patients. Subclinical atherosclerosis was evaluated by carotid ultrasound. *Omentin* rs12409609, in linkage disequilibrium with a polymorphism associated with CV risk, was genotyped in 385 patients and 84 controls. Serum omentin levels were also determined. *omentin* mRNA expression was assessed in a subgroup of individuals. Serum and mRNA omentin levels were lower in axSpA compared to controls. Low serum omentin levels were related to male sex, obesity, inflammatory bowel disease (IBD) and high atherogenic index. rs12409609 minor allele was associated with low *omentin* mRNA expression in axSpA. No association was observed with subclinical atherosclerosis at the genetic or functional level. In conclusion, in our study low omentin serum levels were associated with CV risk factors in axSpA. Furthermore, rs12409609 minor allele may be downregulating the expression of *omentin*. These data support a role of omentin as a CV risk biomarker in axSpA.

## Introduction

Axial spondyloarthritis (axSpA) is a chronic inflammatory disease that mainly affects the spine and pelvic joints. axSpA patients can also show a broad range of disease manifestations, including arthritis, enthesitis, dactylitis, psoriasis, uveitis and inflammatory bowel disease (IBD), among others. All this substantially affects the patient’s quality of life and has important socioeconomic consequences^1^.

Besides, similarly to other chronic inflammatory rheumatic diseases, axSpA is also associated with a higher incidence of hypertension, obesity, dyslipidemia and smoking habit, which are considered classic risk factors for the development of cardiovascular (CV) disease. Moreover, the inflammatory status present in those patients further enhances their CV risk^2–4^. In fact, CV disease is the main leading cause of mortality in patients with axSpA^4^.

In most of the cases, the first step in CV disease is the development of atherosclerosis. This process begins with damage to the vascular endothelium, triggered by the CV risk factors above mentioned, which can lead to structural changes in the vascular wall and that can be detected at the subclinical phase by non-invasive technologies such as carotid ultrasound^2,5,6^. In this regard, it has been shown that SpA patients develop thickening of the arterial wall and formation of carotid plaques more commonly than matched controls^5,7^. These morphological findings constitute surrogate markers of subclinical atherosclerosis^5,7^.

In this pathological context, axSpA patients usually exhibit a dysregulation of certain molecules such as adipokines, metabolic syndrome-related biomarkers and biomarkers of endothelial cell activation and inflammation, contributing thereby to the increased CV risk in axSpA^8^. This is why it is so important to find biomarkers that may help to predict CV risk in these patients.

Omentin (also known as intelectin 1) is a novel pleiotropic adipokine mainly secreted by visceral adipose tissue, endothelium, vascular smooth muscle, colon and small intestine, that not only exerts anti-inflammatory functions, but that has also been described as an anti-atherosclerotic and cardioprotective molecule, among other roles^9^. In particular, it has been demonstrated that omentin enhances insulin signal transduction and glucose uptake, attenuates vascular inflammation, has vasodilatory effect on blood vessels, suppresses reactive oxygen species production and calcification in vascular smooth muscle cells, inhibits foam cell formation and also promotes the polarization of macrophages towards the anti-inflammatory M2 phenotype^9,10^. Moreover, it has been reported that omentin plays a key role in bone homeostasis, inhibiting the anti-osteoblastic and pro-osteoclastic effect of activated macrophages^11^. Low levels of this protein have been associated with metabolic dysfunction (insulin resistance, diabetes mellitus (DM) and metabolic syndrome) and CV disease^12–15^. However, all these studies were performed in conditions different from axSpA such as obesity, coronary artery disease (CAD), DM and rheumatoid arthritis (RA)^12–16^.

Taking all this into consideration, in the present study we aimed to evaluate, for the first time, the genetic and functional implication of omentin in CV risk and subclinical atherosclerosis in a large cohort of axSpA.

## Methods

### Patients and controls

All the experiments involving humans and human blood samples were carried out in accordance with the approved guidelines and regulations, according to the Declaration of Helsinki. All experimental protocols were approved by the Ethics Committee of clinical research of Cantabria (for Hospital Universitario Marqués de Valdecilla, Santander, and Hospital Comarcal de Laredo, Laredo), Ethics Committee of clinical research of Complejo Hospitalario Universitario de Canarias (for Hospital Universitario de Canarias, Santa Cruz de Tenerife), Ethics Committee of clinical research of Madrid (for Hospital Universitario de la Princesa, Madrid), Ethics Committee of clinical research of Elda (for Hospital General Universitario de Elda, Alicante), Ethics Committee of clinical research of Ciudad Real (for Hospital General Universitario de Ciudad Real, Ciudad Real) and Ethics Committee of clinical research of Hospital Universitario de Gran Canaria Dr. Negrín (for Hospital Universitario de Gran Canaria Dr. Negrín, Las Palmas de Gran Canaria). Informed written consent was obtained from all subjects.

385 Spanish patients who fulfilled the Assessment of SpondyloArthritis international Society (ASAS) classification criteria for axSpA^17^ were recruited for this study at Hospital Universitario Marqués de Valdecilla (Santander), Hospital Comarcal de Laredo (Laredo), Hospital Universitario de Canarias (Santa Cruz de Tenerife), Hospital Universitario de La Princesa (Madrid), Hospital General Universitario de Elda (Alicante), Hospital General Universitario de Ciudad Real (Ciudad Real) and Hospital Universitario de Gran Canaria Dr. Negrín (Las Palmas de Gran Canaria). None of them had DM or chronic kidney disease. 84 Spanish healthy controls (who did not have history of CV events or chronic inflammatory diseases) were also recruited for the comparative analysis of omentin (at the genetic and functional level) between axSpA patients and controls.

Data on sex, age, body mass index (BMI), blood pressure, total cholesterol, high-density lipoprotein (HDL)- and low-density lipoprotein (LDL)-cholesterol and triglycerides at the time of study, as well as history of traditional CV risk factors (smoking, obesity, dyslipidemia and hypertension) were collected. Obesity was defined if BMI (calculated as weight in kilograms divided by height in squared meters) was ≥30. Individuals were considered to have dyslipidemia if they had hypercholesterolemia and/or hypertriglyceridemia (defined as diagnosis of hypercholesterolemia or hypertriglyceridemia by the individuals’ family physician, or total cholesterol and/or triglyceride levels in fasting plasma being>220 and>150 mg/dL, respectively). In those individuals with total cholesterol between 200 and 220 mg/dL, a diagnosis of dyslipidemia was considered if the atherogenic index (AI) (total cholesterol/HDL-cholesterol) was ≥4. Individuals were diagnosed as having hypertension if blood pressure was>140/90 mmHg or if they were taking antihypertensive agents. Routine laboratory parameters such as C-reactive protein (CRP) and erythrocyte sedimentation rate (ESR) were assessed at the time of the study. The main demographic, clinical, laboratory and CV disease-related data of patients are displayed in Table 1.

**Table 1:** Demographic, clinical, laboratory and cardiovascular disease-related data in patients with axSpA.

Variable	axSpA (n = 385)
**Men/Women, n**	246/139
**Age (years), mean** ± **SD**	46.3 ± 12.1
**Age at axSpA diagnosis (years), mean** ± **SD**	37.1 ± 10.7
**History of classic cardiovascular risk factors, %**	
Smokers	48.6
Obesity	19.0
Dyslipidemia	24.0
Hypertension	19.5
**Body mass index (kg/m**^**2**^**), mean** ± **SD**	26.3 ± 4.4
**Systolic blood pressure (mm Hg), mean** ± **SD**	126.9 ± 15.4
**Diastolic blood pressure (mm Hg), mean** ± **SD**	78.6 ± 10.1
**Total cholesterol (mg/dL), mean** ± **SD**	191.8 ± 36.8
**HDL-cholesterol (mg/dL), mean** ± **SD**	54.9 ± 16.1
**LDL-cholesterol (mg/dL), mean** ± **SD**	116.6 ± 32.6
**Triglycerides (mg/dL), mean** ± **SD**	104.9 ± 55.4
**Atherogenic index (total cholesterol/HDL), mean** ± **SD**	3.7 ± 1.0
**Atherogenic index** ≥ **4, %**	39.3
**CRP (mg/L), mean** ± **SD**	4.9 ± 9.4
**ESR (mm/1st hour), mean** ± **SD**	12.5 ± 16.7
**BASDAI, mean** ± **SD**	3.8 ± 2.3
**ASDAS, mean** ± **SD**	2.2 ± 1.0
**Anterior uveitis, %**	18.2
**Psoriasis, %**	10.1
**Inflammatory bowel disease, %**	8.1
**Dactylitis, %**	6.3
**History of synovitis and/or enthesitis, %**	67.2
**History of hip involvement, %**	10.5
**Syndesmophytes, %**	34.5
**Carotid IMT (mm), mean** ± **SD**	0.623 ± 0.136
**Carotid plaques, %**	28.9

ASDAS: Ankylosing Spondylitis Disease Activity Score; axSpA: Axial spondyloarthritis; BASDAI: Bath Ankylosing Spondylitis Disease Activity Index; CRP: C-Reactive Protein; ESR: Erythrocyte Sedimentation Rate; HDL: High-Density Lipoprotein; IMT: Intima-Media Thickness; LDL: Low-Density Lipoprotein; SD: Standard Deviation.

Data shown in this table refer to values at the time of the study.

Peripheral blood samples were collected in the fasting state from all the patients and controls at the time of recruitment.

### Carotid ultrasound study

A carotid ultrasound study was performed in all the patients to assess the presence of abnormal carotid intima-media thickness (cIMT) values in the common carotid artery as well as the presence of focal plaques in the extracranial carotid tree (as surrogate markers of subclinical atherosclerosis), as previously reported^7^.

### *Omentin* genotyping

DNA of patients and controls was obtained from peripheral blood using NucleoSpin Blood Kit (Macherey-Nagel, Germany), according to the manufacturer’s instructions. The *omentin* rs12409609 (C/T) polymorphism, in complete linkage disequilibrium with rs2274907 (previously associated with CV risk^18–20^), was genotyped by a pre-designed TaqMan SNP genotyping assay (C___1757143_10) in a 7900 HT real-time instrument (Applied Biosystems, USA), according to the conditions recommended by the manufacturer.

### Serum omentin assay

A commercial enzyme-linked immunosorbent assay (ELISA) kit was used to measure serum omentin levels in axSpA patients and controls (RD191100200R, BioVendor, Czech Republic) according to the manufacturer’s instructions. All samples were analyzed in duplicate and quantified relative to a standard curve, using a 4-parameter algorithm.

### *Omentin* mRNA expression analysis

RNA was extracted from peripheral blood samples of 176 axSpA patients and 27 controls using the NucleoSpin RNA Blood Kit (Macherey-Nagel, Germany), according to the manufacturer’s instructions. Total RNA was reverse transcribed into complementary DNA (cDNA) using iScript^TM^ Advanced cDNA Synthesis Kit for reverse transcription- quantitative real-time PCR (qPCR) (Bio-Rad, USA), and cDNA was amplified with SsoAdvanced^TM^ Universal SYBR® Green Supermix (Bio-Rad, USA). qPCR reactions for *omentin* (target gene) and *GAPDH* (housekeeping gene) were performed in custom 384-well-plates (PrimePCR Assays, Bio-Rad, USA) in a 7900 HT real-time instrument (Applied Biosystems, USA). All samples were assayed in duplicate and experimental control assays were included. The relative *omentin* mRNA expression was analyzed by the comparative Ct method, as previously reported^21^. Normalized values were obtained for each sample and mean values were determined for each study group.

### Statistical analysis

The *omentin* rs12409609 genotype data were checked for deviation from Hardy-Weinberg equilibrium (HWE). Differences in the distribution of genotypes and alleles between patients and controls were assessed by chi-squared test. Strength of associations were estimated using odds ratios (OR) and 95% confidence intervals (CI). The relationship between genotypes or alleles and carotid plaques was tested using logistic regression, while the association with cIMT values was evaluated by ANOVA, in both cases adjusting for potential confounding factors: sex, age at the time of the study and classic CV risk factors (smoking, obesity, dyslipidemia and hypertension). The link between genotypes or alleles with serum levels or mRNA expression of omentin was tested by linear regression, adjusting for potential confounding factors. In all the genetic analyses, the most frequent genotype and allele of *omentin* rs12409609 (CC and C, respectively) was used as reference.

The Shapiro-Wilk normality test was performed and showed that serum levels and normalized mRNA expression of omentin were not normally distributed in our cohorts. Accordingly, these data were log transformed for the statistical analysis. Differences in serum levels or mRNA expression of omentin between patients and controls were assessed by linear regression. The association of serum levels or mRNA expression of omentin with carotid plaques was tested by logistic regression, while the correlation between serum levels or mRNA expression of omentin and cIMT values was performed via estimation of the Pearson partial correlation coefficient (r). The association of serum levels of omentin with categorical and continuous variables was assessed by linear regression and Pearson’s partial correlation coefficient (r), respectively. In all the cases, adjustment was performed for the potential confounding factors above mentioned.

Data were expressed as mean ± standard deviation (SD) for continuous variables, and number of individuals (n) or percentage (%) for categorical variables. Statistical significance was defined as *p* values ≤0.05, and all analyses were performed using STATA® v. 11.1 statistical software (Stata Corp, College Station, TX, USA).

## Results

### Genotype and allele distribution of *omentin* rs12409609

Genotyping success was greater than 98%. *Omentin* rs12409609 genotype distribution was in HWE (p > 0.05). Genotype and allele frequencies of rs12409609 were in agreement with the data of the 1000 Genomes Project for Europeans. In addition, the distribution of the genotypes and alleles of rs12409609 was similar between axSpA patients and controls (p > 0.05) (Table 2).

**Table 2:** *Omentin* rs12409609 distribution in axSpA patients and healthy controls.

Genotype/Allele	Controls (%)	axSpA (%)	OR [95% CI]	p-value
**CC**	48.7	46.9	1 (Reference)	—
**CT**	46.2	44.8	1.01 [0.59-1.72]	0.98
**TT**	5.1	8.3	1.67 [0.54-6.89]	0.35
**C**	71.8	69.3	1 (Reference)	—
**T**	28.2	30.7	1.13 [0.76-1.69]	0.54

axSpA: Axial spondyloarthritis; CI: Confidence interval; OR: Odds ratio.

### Differences in serum and mRNA expression levels of omentin

**Figure 1:**
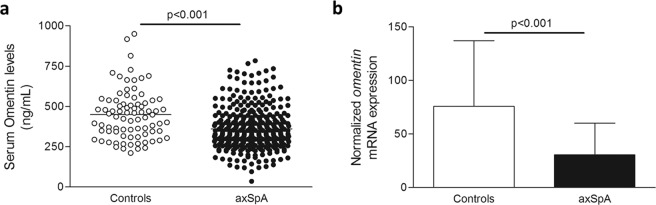
Omentin levels in axSpA and controls. Reduced serum (**a**) and mRNA expression (**b**) levels of omentin in axSpA patients when compared to healthy controls, after adjustment for potential confounding factors. Horizontal bars in Fig. 1a indicate the mean value of each group.

Serum omentin levels were significantly lower in patients than in controls (361.6 ± 129.6 *vs*. 449.5 ± 154.2 ng/mL, respectively, p < 0.001) (Fig. 1a). Interestingly, serum omentin levels were higher in females when compared to males (389.6 ± 150.8 *vs*. 346.0 ± 113.5 ng/mL, in female and male patients, respectively, p = 0.013). Furthermore, the mRNA expression of *omentin* was also nearly 3-fold lower in patients when compared to controls (30.5 ± 29.6 *vs*. 76.0 ± 61.0, respectively, p < 0.001) (Fig. 1b).

### Relationship of omentin with clinical features and CV risk factors

**Figure 2:**
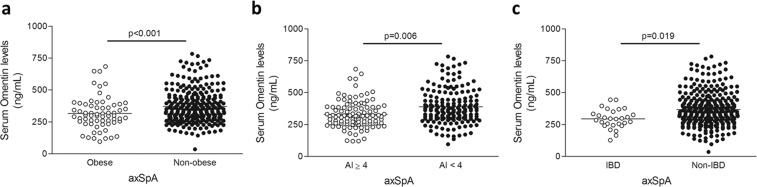
Omentin serum levels and cardiovascular risk factors. Differences in serum omentin levels between obese and non-obese axSpA patients (**a**), axSpA patients showing or not an AI indicative of dyslipidemia (AI ≥ 4) (**b**), and axSpA patients with and without IBD (**c**), after adjustment for potential confounding factors. Horizontal bars indicate the mean value of each group.

We disclosed that low serum omentin levels were associated with obesity and AI indicative of dyslipidemia (AI ≥ 4) in axSpA patients (317.1 ± 126.8 ng/mL in obese *vs*. 372.3 ± 128.4 ng/mL in non-obese patients, p < 0.001, Fig. 2a; 329.5 ± 106.1 ng/mL in patients with AI ≥ 4 *vs*. 389.6 ± 136.9 ng/mL in patients with AI < 4, p = 0.006, Fig. 2b). In addition, we observed that patients with IBD also exhibited lower serum levels of omentin (295.2 ± 76.1 *vs*. 367.7 ± 132.0 ng/mL in patients with and without IBD, respectively, p = 0.019) (Fig. 2c).

### Influence of rs12409609 on serum levels and mRNA expression levels of omentin

**Figure 3:**
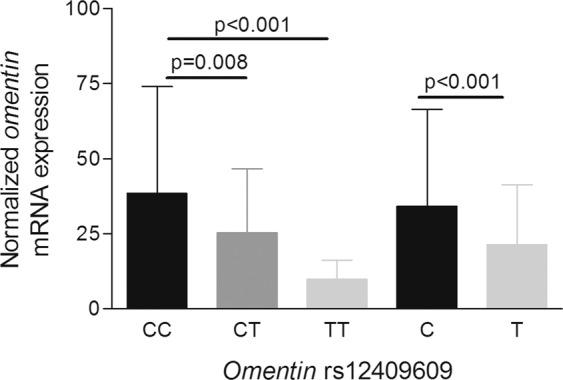
*Omentin* mRNA expression levels and rs12409609. Association of *omentin* rs12409609 minor (T) allele with low *omentin* mRNA expression in axSpA patients, both when genotypes and alleles were assessed. p-values displayed were obtained after adjustment for potential confounding factors.

We found that patients homozygous for the minor allele of rs12409609 (TT genotype) showed the lowest mRNA expression level when compared to those carrying the CC genotype (9.9 ± 6.4 *vs*. 38.4 ± 35.7, respectively, p < 0.001), while patients bearing the CT genotype displayed intermediate mRNA expression levels (25.3 ± 21.4, p = 0.008) (Fig. 3). Likewise, it was disclosed that the T allele was associated with low gene expression of *omentin* in our patients (21.4 ± 20.0 in patients bearing the T allele *vs*. 34.2 ± 32.2 in those carrying the C allele, p < 0.001) (Fig. 3). No statistically significant association was observed between rs12409609 alleles and genotypes and serum levels of omentin (p > 0.05).

### Association of omentin and surrogate markers of subclinical atherosclerosis

No statistically significant association was observed between omentin and markers of subclinical atherosclerosis (presence of carotid plaques and abnormal cIMT values) at the genetic or functional (protein and mRNA) level (p > 0.05).

## Discussion

CV disease is the leading cause of mortality in axSpA patients^4^. Accordingly, it is of main clinical relevance to find non-invasive blood biomarkers that, along with clinical features and imaging techniques, could help us to identify axSpA patients at risk of CV events. For this purpose, in this study we aimed to assess the implication of omentin, a novel adipokine, in the CV risk and subclinical atherosclerosis of axSpA patients. In the last years this molecule has attracted an increasing interest and, consequently, several studies were performed to evaluate the role of omentin in diseases different from axSpA^12–16^. However, despite the clinical importance of the CV risk in axSpA, to the best of our knowledge, this is the first study aimed to address this issue in this chronic inflammatory disease.

In the present study we found that serum levels of omentin were reduced in axSpA patients when compared to controls. This is in line with previous studies performed in DM, CAD and psoriasis, in which decreased levels of omentin were described in such conditions^13,22–27^. Additionally, we noted that the serum levels of omentin were higher in women than in men. This finding on the sexual dimorphism of omentin had been previously described, and was associated with the different pattern of body fat distribution in women and men, as well as with a potential influence of sex hormones (such as testosterone) on the regulation of omentin^28^.

In relation to the mRNA expression of *omentin*, the studies performed so far were mainly focused on assessing the potential difference in its expression in adipose tissue between several diseases and healthy controls. With respect to this, it has been described that low *omentin* expression levels were observed in patients with inflammatory conditions such as CAD, obesity or polycystic ovary syndrome^12,13,29^. In keeping with these observations, in our study we disclosed a decreased mRNA expression of *omentin* in whole blood in axSpA when compared to controls, similar to the results we obtained at the serum level.

As for its anti-atherogenic function, similar to the results obtained in RA by Robinson *et al*.^16^, in our study we did not find any direct association between omentin and surrogate markers of subclinical atherosclerosis (presence of carotid plaques and abnormal cIMT values) in axSpA. Notwithstanding, an association between low serum levels of omentin and CV risk factors such as obesity and AI indicative of dyslipidemia was observed in our axSpA patients. This is in accordance with previous reports from studies performed in the general population and in DM patients, in which a negative correlation between omentin levels and obesity was described^12,30–33^. In this regard, it was proposed that molecules produced as a result of obesity-induced chronic low-grade inflammation may be modulating omentin levels^10,12^. Likewise, in line with our results, other authors reported an inverse association of omentin levels with adverse lipid profiles in conditions different from axSpA^12,31,33^. High AI constitutes a CV risk factor that contributes to the development of atherosclerosis^34^.

In addition, we also disclosed low serum levels of omentin in patients with IBD. This is in agreement with previous reports that showed that serum omentin is decreased in patients with active Crohn’s disease and ulcerative colitis, two types of IBD^35,36^. Accordingly, serum omentin has been suggested as a potential marker of IBD disease activity^10,35,36^. Interestingly, in line with these results, the expression of omentin in the colonic tissue of patients with active Crohn’s disease was also reported to be downregulated^35^. All these data suggest that the lower levels of omentin observed in IBD patients reflect their inflammatory state, both systemically and locally, supporting its anti-inflammatory function. Importantly, recent studies indicate that IBD could be associated with a higher CV risk^37^. This may be due to the oxidative stress and high circulating levels of pro-inflammatory molecules that are the result of the systemic inflammation observed in IBD patients, and that might contribute to the development of endothelial damage and atherosclerosis^37,38^.

At the genetic level, we genotyped rs12409609, a genetic variant located in the downstream regulatory region of *omentin* and that is in complete linkage disequilibrium with rs2274907, a missense polymorphism of the gene previously associated with CV risk^18–20^. In our study we did not observe any relationship between rs12409609 and axSpA nor with subclinical atherosclerosis in these patients. Similar results were obtained in inflammatory conditions such as psoriasis, DM or rheumatoid arthritis^23,39,40^. Interestingly, however, we noted that the minor allele of *omentin* rs12409609 was linked to a reduced mRNA expression of *omentin*, being this association dose-dependent. In this regard, patients bearing a single or double dose of the minor allele (T) showed intermediate or the lowest omentin expression levels, respectively, when compared to those patients homozygous for the most frequent allele (C). Nevertheless, further replication studies are needed to confirm the influence of this polymorphism on *omentin* mRNA expression.

Based on our data and on those reported in previous studies, it seems clear that changes in omentin levels are implicated in the pathology of conditions related to inflammation and cardiovascular disease, given its pleiotropic functions. However, the cross-sectional design of our study precludes drawing conclusions about whether the lower levels of omentin observed in our axSpA patients are the cause or the consequence of their high CV risk. In fact, the results from two prospective studies performed so far on the predictive role of omentin in CV disease are controversial. One of them proposes that low levels of omentin predict the outcome of adverse cardiac events in patients with hypertrophic cardiomyopathy^41^, while the other reports that high omentin predicts CV events in coronary patients^42^, possibly as a compensatory anti-inflammatory and cardioprotective mechanism. Therefore, further longitudinal studies in this regard are warranted.

In conclusion, our data provide evidence for the first time that omentin is linked to obesity and adverse lipid profiles in axSpA. Additionally, low serum levels of this protein are associated with the presence of IBD in axSpA. Furthermore, our results suggest that the minor allele of *omentin* rs12409609 may be downregulating the expression of this gene in whole blood of axSpA patients. Taking all this into consideration, our data support the role of omentin as a CV risk biomarker in axSpA. Accordingly, omentin may constitute a promising target for future therapeutic strategies aimed to increase its circulating levels and, thereby, prevent the development of CV disease in axSpA patients. This would not only lead to a beneficial effect on CV risk but also on features typical of axSpA such as inflammation and bone remodeling, further supporting the relevance of this molecule at the clinical level.

## Data Availability

All data generated or analysed during this study are included in this published article.
